# A Qualitative Study of Autism Policy in Canada: Seeking Consensus on Children’s Services

**DOI:** 10.1007/s10803-015-2502-x

**Published:** 2015-06-24

**Authors:** Cody A. Shepherd, Charlotte Waddell

**Affiliations:** Faculty of Health Sciences, Children’s Health Policy Centre, Simon Fraser University, 2435-515 West Hastings Street, Vancouver, BC V6B 5K3 Canada

**Keywords:** Autism policy and services, Children and families, Canada, Qualitative research

## Abstract

Canadian autism policy has been unusually contentious, with parents resorting to litigation to secure services for their children in several provinces. To ascertain whether consensus was possible on improving services, we conducted an in-depth qualitative interview study with 39 parents, policymakers and researchers across the country. Parents vividly described the stresses of caring for their children, with considerable sympathy from researchers. Policymakers in turn struggled to balance the needs of all children. Yet participants agreed on the need for more comprehensive services across the spectrum and throughout the lifespan, and on the need to “do more for all” children. Our findings suggest that there is an emerging consensus on improving autism services in Canada—which should greatly benefit children.

## Introduction

Public debates about autism policy are hardly unique to Canada. But they have been particularly acrimonious here—encompassing over a decade of conflict between parents of children with autism and provincial policymakers, starting in the late 1990s. As this conflict escalated, parents launched successive legal challenges against several provincial governments, seeking to entrench funding for early autism interventions (Greschner and Lewis 2003). An early precedent in Alberta, where courts ordered the province to expand services for children with developmental disabilities to also include autism, was swiftly followed by the *Auton* case seeking early intensive behavioural interventions (EIBI) funding for preschool children in British Columbia (BC), and the *Wynberg*-*Deskin* case seeking supports for school-aged children in Ontario (Manfredi and Maioni 2005). The conflict peaked when the *Auton* case reached the Supreme Court of Canada, where parents argued that the failure to fund EIBI constituted discrimination under the *Canada Health Act* and the *Canadian Charter of Rights and Freedoms,* with many autism and disability organizations intervening on behalf of the *Auton* families and most provinces intervening on behalf of the BC Government (*Autonv*. British Columbia 2004; Manfredi and Maioni 2005). However, the Supreme Court dismissed the parents’ case, noting that within Canada’s universal healthcare insurance system, children with autism had the same access to core healthcare services as other children—essentially preserving provincial governments’ authority over public funding for health and social services (Manfredi and Maioni 2005).

Facing intense media scrutiny and ongoing parent advocacy, most provinces nevertheless responded with increased funding for autism services, particularly for young children (e.g., Auditor General of Ontario 2013; BC Ministry of Children and Family Development 2015). Yet consistent with Canadian federalism, each province developed its own approach (Motiwala et al. 2006; Perry et al. 2008; Smith et al. 2010). Early intervention services now vary along three dimensions: (1) the relative mix of public and private funding; (2) the degree of integration across services; and (3) the extent of population coverage (Volden et al. 2015). For instance, Nova Scotia provides public intervention services to all young children with autism, although with delays between diagnosis and access. Ontario also provides public intervention services for all “severely” affected preschool children, but wait lists often preclude timely access. Both BC and Alberta provide individualized funding directly to families to subsidize eligible private interventions—with BC’s program being autism-specific and Alberta’s being integrated with financial supports for all developmental disorders. Meanwhile, Quebec has opted to integrate all developmental disorder services—within the public sector. Most Canadian children with autism now receive some combination of early behavioural as well as developmental interventions, e.g., speech-language therapy, albeit with varying intensity (Volden et al. 2015). Across all provinces, parents nevertheless remain responsible for covering any service shortfalls. In response to these marked inter-provincial differences and ongoing shortfalls, parents have called for a national autism strategy, with support from the Senate of Canada (Eggleton and Keon 2007). However, these calls have yet to be heeded.

In Canada, as in many countries, parents have long been influential in raising public awareness about autism and spurring new research on prevalence, diagnosis and intervention (Orsini and Smith 2010; Silverman 2012). Population-based prevalence estimates have also gradually risen (Elsabbagh et al. 2012), while widely-publicized figures from United States (US) monitoring programs have shown that as many as 1.4 % of middle-school children have a clinical diagnosis of autism spectrum disorder (ASD) at any given time (Centers for Disease Control and Prevention 2014). Several factors have likely contributed to these increases, including changing diagnostic criteria and growing public awareness of the social interaction and communication deficits and the restricted interests and repetitive behaviours that characterize autism (Lord and Bishop 2010; Halfon and Kuo 2013). And as more children are diagnosed with autism, more parents are concomitantly affected by unusually high care burdens and costs (Barrett et al. 2011; Kogan et al. 2008). Meanwhile, researchers have responded with numerous studies on EIBI and other treatments (Interagency Autism Coordinating Committee 2012; Warren et al. 2011). (We use the term EIBI to refer to intervention models based on the principles of applied behavioural analysis or ABA, such as discrete trial training or pivotal response treatment). Taken together, these factors have contributed to increased expectations for public services for children with autism.

Public debates about autism policy have nevertheless unfolded distinctively in Canada. The intense conflicts arose, in part, because within Canadian federalism, the provinces are fully responsible for providing health and social services, including education and other support programs for children with mental health and developmental difficulties—including for children with autism. Funds to cover these costs are raised through provincial taxes, with services then being delivered through a variety of models at the provinces’ discretion. The Canadian government also transfers funds, raised through federal taxes, to support the provinces in delivering health and social services, but the federal government has grown increasingly reluctant to exercise oversight over the use of those funds (Maioni 2002; Banting and Myles 2013). For healthcare, however, the *Canada Health Act* still provides an important oversight mechanism. This *Act* outlines the principles that all provinces must follow if they are to receive federal healthcare funding transfers, including ensuring universal access to “medically necessary” services for all Canadians (Government of Canada 1985). What many Canadians have underappreciated, however, is that the *Act* defines “medically necessary” services quite narrowly—comprising hospital and physician services only—thereby omitting many services pertaining to children with autism (Greschner and Lewis 2003). The Supreme Court’s *Auton* decision in essence reaffirmed Canada’s longstanding arrangements that leave health and social programs under provincial jurisdiction. The implication for Canadians is that these services will continue to vary considerably across the country, particularly in an era of waning federal funding and oversight.

As relative outsiders to these vigorous autism debates, but as researchers familiar with the policy process for children’s health and development more broadly, we were concerned that the acrimonious climate could adversely affect children with autism if conflicts could not be resolved. Our goals were twofold: (1) to understand the reasons for the conflicts, particularly in the aftermath of the court cases; and (2) to determine whether consensus was possible, as a basis for improving services for children with autism in Canada. Our overarching aim was to constructively inform policymaking—or the process of making “collective ethical judgments” (Greenhalgh and Russell 2006)—for children with autism. We therefore conducted a qualitative interview study to explore the views and experiences of parents, policymakers and researchers across Canada, and to elucidate disagreements as well as opportunities for agreement among those who had engaged in the autism policy debates.

## Methods

We chose qualitative methods because they are ideally suited for exploring research questions about competing ideas and values, as well as exploring interactions among diverse groups. Given the contentiousness of autism policy in Canada, we also wanted to elicit a wide range of views and experiences. Our purposive sample was further chosen to facilitate a constant-comparative approach to data analysis, derived from grounded theory methods (Boeije 2002; Strauss and Corbin 1998).

### Participants

The sample comprised three groups of knowledgeable participants who had been engaged in the public debates. To recruit our sample, we identified potential participants who had attended relevant public conferences about Canadian autism research and policy, where contact information was publicly available. Furthermore, we accepted recommendations from knowledgeable research and policy colleagues and from participants themselves. We also spoke about our study at public events and asked interested individuals to contact us privately. We then invited potential participants who met the following inclusion criteria to be interviewed. We sought *parents* who had raised children with autism from the time of diagnosis, and who had actively participated in public forums or community organizations at the local, provincial or national levels. We chose not to seek parents of children with a very recent diagnosis, e.g., within the past 2 years, to avoid burdening these families. We sought *policymakers* who held appointed senior civil service positions within provincial governments and who held primary responsibility for autism programs and funding. We chose not to seek federal civil servants due to the responsibility for decision-making regarding autism services residing at the provincial level in Canada. We also did not seek elected officials. As well, we sought *researchers* who specialized in autism, who held university faculty positions in the health or social sciences or related disciplines, and who had participated in public forums or community organizations.

For *parents*, our approach garnered a diverse sample including members of informal parent support groups, formal advocacy groups, and parent-run service organizations, as well as individuals who had participated in various public policy consultations. Our sample also included parents of young people with autism at all ages and developmental stages, from early childhood through early adulthood. For *policymakers*, we garnered a sample of civil servants whose responsibilities included overseeing autism policy and making direct service and funding decisions at the provincial level, with experience ranging from several years to several decades. These civil servants ranged from assistant deputy ministers to executive directors to policy analysts. For *researchers*, meanwhile, we garnered a sample of relatively senior academics who had conducted basic or applied autism research, who had often provided clinical services, and who had engaged in policy debates or provided advice to policymakers.

We conducted an initial round of participant recruitment and interviews in 2007–2008 and a subsequent round in 2011–2012. We completed interviews with 39 participants—15 parents, 13 policymakers and 11 researchers—from eight provinces, including both more and less populous and prosperous regions of Canada.

### Qualitative Interviews

We developed a semi-structured interview protocol, drawing on our previous qualitative studies of the policy process for children (Waddell et al. 2007). Our protocol covered the following topics: (1) how participants came to be involved with autism; (2) participants’ understanding of provincial policies and services for children autism, and what they believed the most important unresolved issues were; (3) participants’ experiences of engaging with the other two groups, and how these interactions had influenced their views; and (4) how all three groups might work together more effectively to improve outcomes for children with autism. Our semi-structured approach allowed us to address our research questions, e.g., ascertaining participants’ views on the impact of the Supreme Court’s *Auton* decision, while also allowing participants to speak at length about additional issues of their choosing. The full interview protocol is available upon request.

Interviews were conducted in person where possible and by telephone otherwise. The first author, an experienced qualitative investigator with a health sciences background, conducted two interviews. The second author, an experienced qualitative investigator and qualified child and adolescent psychiatrist, conducted 13 interviews. The remaining 24 interviews were conducted jointly. Interviews ranged from 60 to 120 minutes, with a mean length of 80 minutes, and were digitally recorded and transcribed verbatim. After the initial round of interviews, we analyzed the data to identify potential themes. We then conducted a subsequent round of interviews with additional participants to further explore and organize these themes, until we reached conceptual saturation.

### Data Analysis

For our data analysis, to identify both conflicts and potential areas of agreement, we employed the constant-comparative approach (Boeije 2002; Strauss and Corbin 1998). In our case, this involved comparing views and experiences within groups (e.g., parents compared with other parents), across groups (e.g., parents compared with policymakers), and across regions (e.g., participants from one province compared with those from another). We conducted our analysis in three stages: (1) coding interview transcripts and establishing preliminary themes through frequent comparisons; (2) recoding the data using the preliminary themes and identifying the most frequent and salient themes; and (3) organizing these themes into overarching categories that addressed the study questions, including the sources of conflict for each group and the points of agreement across groups (Creswell 2013). The categories and themes are presented below together with illustrative quotes, edited to ensure confidentiality (Buetow 2010). At each stage, one author conducted the initial coding before the other reviewed the coding in depth, querying interpretations and challenging assumptions. Triangulation was thereby achieved through the use of multiple data sources (parents, policymakers and researchers from different regions of the country), and through the involvement of two researchers with different areas of expertise in the analysis (Farmer et al. 2006).

## Results

Our findings encompass five overarching categories that emerged from the analysis: (1) implications of litigation; (2) parents in the lurch; (3) policymakers in the crucible; (4) researchers in the mix; and (5) children in the balance. Within each category, we have illustrated the various themes with shorter quotes that represent participants’ diverse perspectives, as well as longer quotes that provide the clearest expression of each theme. Throughout, we have endeavored to faithfully represent all participants’ views and experiences. The Table 1 provides an overview of our findings.

**Table 1:** Overview of qualitative findings on Autism policy in Canada

Implications of litigation
Expressing dissatisfaction with the outcomes of litigation
Arguing for guaranteed funding for autism interventions
Parents in the Lurch
Learning about autism and seeking early intervention
Adopting a pragmatic approach to children’s outcomes
Attesting to the impact of autism on families
Championing their children’s needs
Becoming advocates for *all* children with autism
“Paying it forward” to the next generation
Disagreeing on the best ways to influence policy
Policymakers in the crucible
Coping with the intensity of the public debates
Acknowledging parents as exemplary advocates
Collaborating with parents and researchers
Expressing regret about adversarial and reactive policymaking
Balancing investments in autism and other childhood conditions
Crediting parents with the strategic use of research evidence
Researchers in the Mix
Expressing empathy for children with autism and their families
Developing intervention programs to support children and families
Engaging with policymakers
Using research evidence to inform policy and practice
Children in the Balance
Expanding services across the autism spectrum
Extending autism services throughout the school years
Supporting young people during the transition to adulthood and beyond
Addressing socioeconomic and geographic inequities
Providing more comprehensive services
Doing more for *all* children

### Implications of Litigation

#### Expressing Dissatisfaction with the Outcomes of Litigation

All participants were asked to reflect on the impact of the high-profile Canadian court cases. While autism services had “done nothing but grow” in the wake of the litigation, particularly EIBI for young children, no one was wholly satisfied with the outcomes. Parents acknowledged that “if it wasn’t for those families behind *Auton*, who knows whether we’d have any supports at all.” Yet many remained dissatisfied: “Prior to the litigation there was no autism funding of any consequence and subsequent to the litigation there was, but I was very disappointed in the Supreme Court.” Meanwhile, researchers expressed admiration for the families who led the litigation, yet also expressed concern: “They did an amazing thing, so I really believe that they’re heroes, but the tragedy is that they feel like they lost.” The main source of dissatisfaction was the way that new intervention programs were implemented, with many citing problems such as waitlists in provinces with publicly-provided services, and parent burden in provinces with individualized funding for private services. As one parent rued: “This whole process that we have for autism in the province is flawed, and it needs a good hard look.” Policymakers agreed: “If we could go back and redo this whole thing, we’d do it very differently.”

*Parent:* The policy changes and the programs that have developed were primarily the result of litigation and that’s not a really good way of developing policy. A lot of the problems we’ve had are because this wasn’t developed through policymakers sitting down and thinking about how they could work out a good program. This was the result of litigation.

*Policymaker:* We had to move very quickly to get services out the door. There wasn’t a lot of time for thoughtful, careful consideration and dialogue with experts in the area as to strategies that we could put in place that would be effective for kids. So another challenge for us now is taking a step back, taking a breath and saying, we may not be totally pleased with what’s happening, but it gives us an opportunity to revisit some of those policy decisions that were made because of political pressure.

#### Arguing for Guaranteed Funding for Autism Interventions

Parents also feared that autism service gains would not endure: “My concern is that a few years from now, we’ll have a change of government and *whoosh,* the money’s gone and the commitment to intervention is gone.” They had hoped that the court cases would provide more certainty: “Without them being enshrined in health legislation, there’s no guarantee for families that the services are going to be there tomorrow.” Some parents further argued that the lack of guaranteed public funding for autism interventions violated the implicit promise of Canadian healthcare to protect them from the catastrophic costs of unexpected health problems. The result, they said, was a “two-tiered system,” because with “autism on the severe end, it’s prohibitively expensive to go a private route.” Many participants found this situation fundamentally unfair: “It’s not reasonable or possible to expect some groups to go through the court system to remedy such injustices.”

*Parent:* If you’re autistic or if you have a child with autism, your core health needs in this country are not being met. It’s very straightforward. It’s a question of right or wrong.

*Parent:* We’re not asking for someone to hold our hand, but up until 5 years ago when our son was diagnosed, it was my understanding that if you got sick in Canada, you got taken care of.

### Parents in the Lurch

#### Learning About Autism and Seeking Early Interventions

To better understand the conflicts from the parents’ perspectives, these participants were asked about their first encounters with autism. They typically began by recounting how the initial “shock” of the diagnosis rapidly shifted to a search for answers: “Getting the diagnosis meant absolutely nothing to me, I didn’t know what autism even meant, but that began my quest to understand as much as I could to help him.” Many parents spoke of a “panic” to secure interventions during the “critical time period after the diagnosis” because “with children who are deeply, deeply in the middle of autism, it really does seem to be that the longer left untreated, the worse it gets.” The sense of urgency was heightened by the possibility that early interventions could significantly improve children’s outcomes: “I was told there was a window that was closing.” As one researcher commented: “It’s like being told your child has diabetes but you can’t have the insulin.”

*Parent:* We’re told we need to do something, we need to do it very intensively, and we need to do it soon. So parents are left with a real vulnerability. Maybe they’ve been told that, say, ABA is the way to go, because that one’s got the most science behind it. So there’s this incredible burden. Here’s this diagnosis. I’m not going to get government help. I’m told this is what I ought to do. It’s 40 to 60 to 80 thousand dollars a year. But if I don’t do it, I’m somehow depriving my child.

#### Adopting a Pragmatic Approach to Children’s Outcomes

On the interventions themselves, parents broadly agreed that everyone “just wants the best for their children.” But they diverged over the prospect of “recovery” from autism. Some were confident that “kids caught early and treated correctly can pretty much recover.” One asserted: “You *can* lose your autism, I know people say you can’t, but my second child did, because we found out at 12 months and intervened.” However, others expressed reservations: “Are you really saying you’d like to eradicate autism? What does ‘cure’ mean? Is that the end of the quirky wonderful gifts that autistic individuals have given to society?” In the end, most parents endorsed a pragmatic approach.

*Parent:* I’m hoping that with my oldest boy, intervention is going to provide him with the ability to eventually live independently and support himself, and if he can do that, we’ll be delighted. Now my youngest son, no, he’s going to need supervised, supported care all of his life. He has a 40-hour-a-week intensive program, he’s making huge gains, but he still has no cognitive understanding of danger, he has a lot of echolalic language, most people don’t understand what he’s saying. But he can dress himself, he ties his shoes, he can use the toilet. That’s what intervention has done for him. Without intervention, he’d still be in a diaper.

#### Attesting to the Impact of Autism on Families

As children’s lives unfolded, parents faced mounting challenges: “I have children on either end of the spectrum. I have a child who’s very classic and has a lot of issues. Then I have a child who has Asperger’s. It’s a little nuts here at times [laughing].” Their child’s needs affected every aspect of their lives, from finances to relationships to parenting capacity: “It’s very, very hard being a single parent raising a child with autism. Now I see how my other child took a back seat, even with my best intentions.” However, it was the struggle to secure services that affected them most acutely: “We’re tired, we’re battered, but it’s easier to do it by yourself, because battling the system, you’re done.”

*Parent:* The marriage so far? We’ve celebrated 20 years already. But I think of all the things we neglect, because autism has put that heavy blanket on the family, and it’s taken us down. We don’t get to travel, because we can’t afford it. We don’t get to do this or that, because financially we’re not there, or it’s not going to be the best fit for my daughter.

*Parent:* The child with autism takes up so much energy. You’re fighting these different systems that are supposed to be there to help you. You’re trying to work all these extra hours to do some type of therapy or intervention. You never go to bed at night, ever, thinking, “I did enough today.”

#### Championing Their Children’s Needs

Every parent talked at length about how they devoted much of their time to obtaining supports and educating others about their child’s needs: “You need to speak for your child until they can speak for themselves.” As de facto “case managers”, parents felt singularly responsible for securing services: “It does seem to fall on the parents for the most part to make these changes happen.” They often found it “overwhelming” to have to judge which services were best for their child: “I was just like a blank blackboard, I needed to learn all of this stuff and even then I had a hard time keeping out what wasn’t sound and what was.” As one parent explained: “At the end of the day, all of those professionals are going to move on, but you still need to build your child for life.” Policymakers also acknowledged: *“*I have a huge respect for these parents when I hear some of what they’re living with. They would do just about anything for their children.”

*Parent:* The social workers didn’t know where to send him and at that point they were saying, maybe we could have him charged with an offense, then he would be eligible for the forensic institute. Well, you can imagine, because he was cognitively like a little child, he was my baby, although he had horrendous behaviour. And so we used every strategy we could to try to get him a place where they understood how to support him and give him the treatment that he needed.

#### Becoming Advocates for All Children with Autism

Despite the burden of caring for their own children, many parents went on to become dedicated advocates for *all* children with autism: “When families feel that they’re confronted by a system which is not functioning the way they want, you advocate for what you believe are the rights of the child.” Some developed this capacity as their own child made progress: “I had more time to give to other families.” Others made sacrifices: “For the past 5 years, nothing else has been addressed in my life except my work as an advocate. My house is falling apart [laughing].” They were driven by the ongoing need to campaign for autism services for everyone: “Those families at the beginning of their voyage need the support as much as we did.”

*Parent:* The group of parents who had been running the organization before me had been quite vocal and had pushed open the doors with government. So I just kind of stepped in and said: “Okay, we’re here, and you need to help us!” If an opportunity presented itself and people wanted to know about autism, we were there. We just started small, we got the ear of a few political party members, and the government then decided to start early intervention.

#### “Paying it Forward” to the Next Generation

These parents typically described their lobbying efforts to establish and improve autism services and supports as an investment in the future: “What I’ve been doing is to advocate systems change, because that in turn helps my children and it helps the children that follow.” In essence, these more experienced parents were “paying it forward”—giving to others with no expectation of direct reciprocation. As one parent noted: “I was doing it for my son and all the other children that are like him, and those who aren’t as high-functioning as him, and those who are higher functioning than him, but seem to be invisible.”

*Parent:* That was the reason for me, organizing social events where people would come and they’re like, “I’ve never seen another child with autism,” so they thought that their life was so isolating and alone, and they just couldn’t imagine anyone else was living this life. So when they saw that they weren’t that different from this huge group of people here, it gave them a sense of community, which was really nice.

*Parent:* It started out very selfishly, obviously. When his pediatric neurologist first told us that he thought our son might be autistic, well, I was really disgusted by the complete lack of services in this province and in this city in particular. There was just nothing here. And so, early intensive intervention being the key, we started to work very furiously trying to get government to do something for us. It’s probably not going to do our son any good. But we still try to do as much as we can.

#### Disagreeing on the Best Ways to Influence Policy

Yet parents ultimately disagreed on the best ways to influence policy, espousing differing strategies for activism. Some set out to “collaborate or die”: “I wish I could tell you our message was so fantastic that people listened to us, but the reality is that politics is a game of relationships.” Yet others were skeptical of collaborating with policymakers and took a more adversarial approach: “They’re not truly representing the parents, they’re receiving funding from the government.”

*Parent:* We have to work in cooperation with the government, so we try to do this in a positive manner. Some of the families do not find that to be the fastest way to see change happen, so there are some more vocal families that are very upset.

*Parent:* I don’t know why they’re completely satisfied with what they’re getting. They’re not getting anything, and they’re not helping their children. I don’t understand it. I don’t know why we all can’t get together and demand services. You know, the squeaky wheel gets the grease.

### Policymakers in the Crucible

#### Coping with the Intensity of the Public Debates

When policymakers first became involved with autism, they were often taken aback by the intensity—if not outright acrimony—of the public debates in the field. Some approached it as “an interesting and challenging file, a puzzle to resolve.” However, many had longstanding commitments to children with autism: “I began working with children with autism on the frontlines right after high school, starting as a one-on-one worker with a young man with ASD, then I worked my way up into greater responsibility.” Therefore, these policymakers felt “badly burned by those parents who have been incredibly adversarial.”

*Policymaker:* I was a special needs worker with responsibility for kids with autism. I worked very hard to make an inclusive setting for all kids with disabilities in our area. One of my key families had a child with autism who had totally exhausted all the resources we could offer and the family was at the end of their rope. And the only resource available was an institution. That’s when I decided that there needed to be alternatives, so I came into policy.

*Policymaker:* We care deeply about what we do or we wouldn’t be here.

#### Acknowledging Parents as Exemplary Advocates

Policymakers noted that “the level of advocacy in autism is really quite remarkable”, compared with advocacy for other childhood conditions. They particularly noted the impact on public perceptions: “Way back, there was this public view that somehow parents were at fault, but the autism advocates have created a more sympathetic picture.” At the same time, many policymakers had also experienced “absolute scrutiny, with a microscope on everything we’ve done from certain families who believe strongly in one way.” One reflected: “The noise about litigation has died down, but people in government have fairly long memories about what was a hot seat.” However, almost all policymakers readily acknowledged that “at the end of the day, sometimes being noisy and strident works.” Indeed, it was exactly this approach that had led to increased intervention funding: “It started because of an extremely well-organized group with some very determined people who were able to assemble a group of parents and inspire them.” Some policymakers even described these “articulate, committed” parents as *exemplars* for effectively influencing policy.

*Policymaker:* This situation is sometimes attributed to parents advocating, as though somehow advocating is unfair. Well, in fact, this is more a lesson to people on *how* to advocate.

#### Collaborating with Parents and Researchers

In this challenging environment, policymakers nevertheless tried to proactively address parents’ concerns whenever possible. Some were confident that they had made a difference: “I’m very proud of our treatment program. I think the kids involved get a really excellent program and get a very good start at school.” In some provinces, policymakers had also successfully collaborated with parents and researchers on the development of new services: “It’s a wonderful program, one that is really family friendly.” Parents in these particular provinces agreed that policymakers were “reasonable people, working hard” who “feel that they’re doing the right thing for families in the long run.” As one researcher added: “I think policymakers felt on the hot seat and I think some of them were really upset with some of the stories they were hearing. I mean, some parents came in and really tried to communicate to them what their life was like, so I think things have turned around.”

*Policymaker:* We knew across the country that court cases were happening. I think the government knew that they could introduce something or something would be imposed upon them. We had a very sympathetic minister at the time, just an incredibly good guy, and he certainly met with the families and was very touched by their experiences and really fought hard to get the funding for the program. I think he feels quite good about having done it and I think the families were quite pleased that he really, truly listened to them and did do something for them.

#### Expressing Regret About Adversarial and Reactive Policymaking

Policymakers nevertheless expressed considerable regret over the adversarial process that had prevailed in many provinces. In particular, they regretted making decisions reactively, “rather than thinking about what we can do more proactively.” Whenever possible, they preferred to take a more diligent approach, arguing that rushed decision-making led to poor outcomes: “I have significant problems with the autism service delivery system, the way it’s structured right now, it’s something I inherited, and if we were to start from square one, I wouldn’t even begin to approach it that way.”

*Policymaker:* In a very, very short timeframe—which I think is irresponsible—we had to come up with a program according to the ideal views of a few people who were connected, without any time to think about the risks and pitfalls, and no time to really ground it in research. It was done in reaction to courts. Our program wouldn’t even exist if it wasn’t for the lawyers saying, “We think there’s going to be a challenge, you better do something.” That’s the wrong way to make social policy.

*Policymaker:* It’s a double-edged sword having such a strong and well-organized parent lobby. On the one hand it’s a very small group of kids relative to the full range of other kids with very complex problems that we deal with in our ministry, but they certainly have the greatest share of the headlines, and it creates a lot of attention and action. The downside, though, is that sometimes that action is more reactive than strategically responsive. So it would be great if the parents and the policymakers were actually spending more time face to face and working together rather than being adversarial.

#### Balancing Investments in Autism and Other Childhood Conditions

In keeping with their broader responsibilities, policymakers struggled to balance autism with other children’s mental health and developmental difficulties: “Those are the kinds of discussions that we end up having around our senior table, just trying to come to terms with what’s fair and equitable, since you can’t do it all.” They stressed, “no one is questioning the need.” But some had encountered “resentment that autism has taken up too much government time and money.” Others suggested that the problem was much broader than autism: “We have limited resources, period, so that’s a huge strategic question around government’s role with kids with special needs and to what extent do taxpayers expect governments to fund these services.” Yet as one policymaker noted: “Autism has done better because it’s not part of the children’s mental health system, which is severely underfunded on all fronts.” Another policymaker explained: “If we look at child welfare or youth justice, these are ‘deep end’ kids who typically come from very impoverished backgrounds, so their best lobby is their probation officer or their social worker—very different than the autism lobby that typically is much more affluent, well educated and well organized.”

*Policymaker:* At one point, we realized that we had to do for kids with autism what we would do for other children with disabilities. We were looking at consultation with families and it was recognized that that services for kids with autism were not unique, that there were a number of other groups of children that were not getting the types of services that their families wanted, or that research and best practices told us were effective and needed to be provided. It provided us a unique opportunity to really try and do a coordinated approach that would benefit all kids with disabilities and their families.

*Policymaker:* That will be one of the challenges over the next while. How do we continue to improve programs and services while balancing the attention kids with autism get against services and supports for kids with other kinds of special needs? We have lots of kids who don’t have such dedicated programs.

#### Crediting Parents with the Strategic Use of Research Evidence

Participants also talked of parents shifting the balance by equating autism with physical health problems and aligning it with the “medical paradigm.” As one parent described: “Like we get cancer treatment, get treatment for diabetes, for other illnesses, this is the type of thing that should be treated by healthcare professionals and therefore supported by whatever your provincial healthcare system can afford.” In contrast, policymakers were more likely to equate autism with chronic mental health and developmental difficulties. At the same time, by offering solutions supported by research evidence, parents of children with autism bolstered their case, making it easier for policymakers to reconcile competing claims and to allocate new resources. As one policymaker indicated: “In autism, you pretty much get the same story for what’s needed no matter who you talk to.” Parents also attested to the strategic use of research in influencing policy: “The data is our ultimate protection against politicians deciding that this is no longer a valuable program.” Policymakers further observed that “the researchers don’t mind being in play on this, they’re very attuned to the parents.” For their part, many researchers overtly supported the parents in the policy debates: “This province was completely unprepared, nobody believed us when we told them there’s a huge increase in the number of kids coming along and the best chance we have is early intervention.” Another researcher flatly declared: “The parents are right, there’s nothing for kids with autism in this province, zero, and that’s criminal.”

*Policymaker:* Parents are always compelling. I know, because I’ve sat in on many meetings. When parents meet with ministers, it’s very compelling. But in the advocacy world, it’s helpful if you can move to, “Here’s five things to do and here’s the relative value and payoffs of those things and let’s continue to do the research.” The autism parents talk about the research. The mental health parents don’t, they just talk about getting help, and it’s just not as sophisticated a strategy.

*Policymaker:* The interesting thing about autism is that they’ve been able to demonstrate the need. But also, they’ve demonstrated that there are effective programs and services to meet the need. It’s not just an advocacy that says: “Give me more.” It’s an advocacy that says: “Here are the things that will help my kid, can we get more of those?” The argument for investing where there’s research is far more compelling than just, “give me more and I have no evidence.” I think that’s underappreciated.

### Researchers in the Mix

#### Expressing Empathy for Children with Autism and their Families

Autism researchers never expected to become involved in intense public debates over services. Rather, many started with curiosity-driven research: “From the time I was an undergrad, I was intrigued by this group of people who seemed to be so unusual.” Yet many went on to develop “longstanding relationships” with children and families, often through their clinical encounters: “There’s something incomparable about the experience of teaching a really hard-to-teach kid to do something important, nothing touches it.” They expressed deep empathy for the difficult circumstances faced by parents: “I could just show you family after family after family where the system has completely failed them.” Therefore, these researchers came to feel compelled to “make life better for these children and these families.”

*Researcher:* The intensity of the clinical research that we’re doing, in terms of how close you get to these families, I mean, there are weeks that there’s not a dry eye in the place, because a kid has deteriorated and it’s become really obvious and everybody’s walking around with a huge burden that week.

*Researcher:* One of the things that motivated me getting into research, into the clinical end, originally was the feeling that it really wasn’t ethically responsible to be doing research with this population without being able to give them something back.

#### Developing Intervention Programs to Support Children and Families

To fulfill their sense of obligation to children and families, some researchers went on to strive to improve services: “I proposed a model of treatment and a model of service delivery and tried to get buy-in from everyone in the government and all the clinicians.” Some were directly responding to families’ concerns: “It was totally parent driven, they needed that answer and we did it, 2 years of my life.” Others were responding to “serendipitous” requests from policymakers: “When things started to move in terms of government being lobbied heavily by parents, I was one of the people who ended up being called up.” Researchers ultimately found these experiences to be rewarding: “I feel good helping the province deal with tough issues.”

*Researcher:* I needed another project like a hole in the head [laughing], particularly one on that scale, so it wasn’t necessarily an easy decision to make, but it was such an incredible opportunity and did have the potential to make such a difference.

#### Engaging with Policymakers

Even self-identified “basic scientists” felt a responsibility when policymakers requested their help: “When the ministry calls about autism, I do feel an obligation to share what I know.” On the other end of the spectrum, some highly-engaged researchers consulted directly to politicians on a frequent basis: “There was a personal connection with him and I would be called upon in an informal way once he got into power.” These researchers persevered with policy-oriented research and consulting despite the lack of conventional academic incentives: “I could probably write two more papers a year if I was not doing this.”

*Researcher:* My time spent with the government? I see it as part of my duty to respond to their requests, so that’s what I do. Now if you ask me to evaluate if it’s making a difference, I have no idea. I’m unable to judge that. I hope it does. At least I give them the information I have, which is informed by the science.

*Researcher:* I used to get very pissed off at policymakers for not paying attention to evidence. I thought my responsibility ended at publishing, but that isn’t good enough. You have to listen carefully to policymakers’ questions. It’s not just the evidence that influences their decisions. There are a lot of other things as well. And, guess what? That’s the same thing as me as a clinician. It’s not just the evidence that influences whether I use this treatment for a kid or whatever. So it’s a matter of realizing that we’re all struggling with the same sorts of issues and so don’t be so arrogant.

#### Using Research Evidence to Inform Policy and Practice

On balance, policymakers seemed appreciative of these researchers’ efforts: “The world of academia and research has come an awful long ways in terms of being much more practical and providing more useful information to policymakers.” Some policymakers added that autism policy could go still further in terms of reflecting the available research evidence: “People make policy decisions and practice decisions that are uninformed and I don’t want to see us doing that.” To address this problem, policymakers said, they tried to “lever existing capacity” for research: “We pride ourselves in having developed a very large network of people who we consult with periodically in the academic community.” In provinces with relatively well-established services, policymakers were also starting to use research evidence to adjudicate parents’ claims: “I can’t imagine how else you’d decide how you were going to spend public money, if it wasn’t based in some evidence, otherwise you’d have everybody coming out of the woodwork.”

*Policymaker:* I was appalled at the total lack of evidence that we had around decision-making and around expenditures for these kids and families. My clinical background from 100 years ago [laughing], just the nature of my training, it disturbed me, the invasiveness for families that some of the interventions seem to have, with very little science behind them to say whether they were making any difference or not. We actually could be doing far harm than good.

*Policymaker:* People were going to court saying, “We want this kind of intervention.” So you get caught up in this whole thing. Now, the ministry is trying to develop a common language across the province around what is evidence-based practice, so that there’s a better way to not only talk to parents about that, but to have that terminology better understood throughout the system. From a policy perspective, that’s an important step forward.

### Children in the Balance

#### Expanding Services Across the Autism Spectrum

Despite the intensity of the conflicts, most participants argued for more collaboration around shared goals: “It’s the kids who are suffering when people are fighting this way.” One parent urged everyone to overcome past differences: “We may have argued over how we got there, we may still argue, but as a community we need to understand that it’s only together that we’ll actually make a difference for children.” Indeed, there were several areas of agreement across all participant groups, starting with early intervention. While parents were generally grateful for early intervention—“my son is so much better now, he’s the poster boy”—many expressed concern about the exclusive emphasis on certain EIBI models. As one parent described: “Many children like my own received extensive early intervention to no avail.” Policymakers were similarly concerned: “The government has poured a tremendous amount of money into ABA, and wasn’t all that excited about hearing there were other ways of potentially working with kids with autism.” Ultimately, participants argued for a more diverse array of interventions.

*Parent:* We’re very lucky. We have a wonderfully social, loving son. He’s a wonderful member of our family. We’re lucky that he’s able to participate in our life fully. Most families don’t have that. But what we’ve got is a child who’s completely non-verbal, so teaching my son has been very, very difficult. Every one of these children is so different. There is no one-size-fits-all.

*Policymaker:* I just dream of having researchers and service providers and parents all together just having rich dialogue about where are we going. Okay, these are the resources we have currently, so what could we do differently?

*Researcher:* There are a lot of programs that are not necessarily the best possible program, or not a good match for the specific child and family, so I think the urgency has to be tempered by what’s right for this child in this context.

#### Extending Autism Services Throughout the School Years

Many participants also expressed reservations about the prevailing emphasis on early childhood in general: “The little guys are getting not only all the media but all the money.” Most parents reported a sharp drop in services when their children entered school, compounded by a lack of staff with the training and experience to support students with autism: “The school system is probably the most regressive and unaccountable system that we have left in the province.” Many also reported that social experiences such as bullying and loneliness were particularly difficult for children with autism during the school years. Researchers and policymakers concurred that the transition to school for children with autism could be “terrible” and that the school system was “struggling.”

*Parent:* My son is the bravest person I’ve ever met. He needs to be honoured for getting up every single day and facing what he faces, not really feeling welcomed in the school system his whole life. He says things like, “Nobody liked me when I was little, but now I’m very popular.” But his idea of popular is that kids say “hi” to him. He still comes home from school and he’s alone.

*Policymaker:* One thing we will be dealing with is the boundary between 20 h of service per week for preschoolers, and full time inclusion in a classroom setting when you start kindergarten, and needing to find some ways to blur that boundary, to make sure that there are a broader set of people within the school system who have the basic understanding and skills needed to interact with kids with autism.

*Researcher:* It would be nice to have some continuity between what’s happening with kids in preschool and what the parents are learning then, and what happens to them when they go to school. But there’s a real disconnect between health and education in our province.

#### Supporting Young People During the Transition to Adulthood and Beyond

Beyond the school years, *all* participants voiced concerns that young people were disappearing into a “black hole” of services and supports in adulthood: “I’m completely dumbfounded as to the complete void out there for adults living with autism.” Many parents expressed dread about the transition to adulthood: “Once my son graduates, I’m quite frightened to think how little there will be for him.” Researchers confirmed that “parents are always thinking about the future” because children with autism “are not like typical children where they have increasing autonomy and a wider circle of friends and get involved with the community—if anything, these kids become even more dependent on their families.” Policymakers agreed: “If we’re not going to commit to a lifespan response for these kids and families, I’m not so sure why we’re spending all the money on the front end.”

*Parent:* He’s still dependent on adults to break tasks down for him and help manage his behaviour if he gets overwhelmed. He’s heading into adulthood. How am I going to replicate that in the real world? Wherever he ends up working, he’s going to need an aide, because there was never a classroom that was able to teach him how to be independent.

*Policymaker:* I think it is just criminal that at the age of 18 these kids completely fall off the radar screen and have minimal supports available to them. We spend huge money and time in intervention until the age of 18 and then all of a sudden, it’s like the ship has sailed and you’re on your own in the ocean.

*Researcher:* One of the things we haven’t talked about is adults and what happens to adults. Because it’s a different ministry, different source of funding, they just get nothing compared to what the kids get and the early intervention gets.

#### Addressing Geographic and Socioeconomic Inequities

Inequities were another concern for all participants—both geographic and socioeconomic. In several provinces, policymakers admitted that they still struggled to “make sure that families get the same level of service” in “a smaller community at one end of the province or a bigger community at the other end.” All participants were also acutely aware of the differences across provinces in the level of investment in autism services: “We’re receiving significantly less than any other jurisdiction in the country.” These geographic differences had a particular impact on parents: “If my daughter’s funding is cut off, there is a great possibility that we may have to move.” Beyond this, many participants also told of the “unspoken story about social inequalities in autism”—with high-income families “getting into services almost immediately” or “going down to the US or going to a private psychologist” and low-income families “having to wait months.” Such disparities conflicted with widely held values: “That’s crazy to me, that’s crazy as a Canadian.”

*Parent:* We’re happy and grateful for whatever we can get that government will pay for because we’ll access whatever else we need on our own money and coordinate that. But it just leaves me with a sick feeling whenever I generalize to the whole population of kids and families who are waiting for help.

*Policymaker:* Because we offer service to anyone who’s got autism, we see people that move here from other provinces to get service for their children. And that’s not the way life should be. People shouldn’t have to move to get help.

*Researcher:* I think it’s working fine for some families who can top it up with their own funding without having to put a second mortgage on their house and eat macaroni and cheese for the rest of their lives. It’s working okay for some families because the kid doesn’t need more than a few hours a week or the service provider’s really good. But the families with more impaired kids, for them the funding is a drop in the bucket, or for the immigrant families, or poor families, or Aboriginal families.

#### Providing More Comprehensive Services

Ultimately, all participants broadly agreed on the overriding need for a “more comprehensive set of programs and services for kids at any place on the spectrum” and “throughout the lifespan.” As one participant articulated: “There’s heterogeneity among interventions, there’s heterogeneity among children, and we can match them better to each other.” Participants also broadly concurred that the focus should always be on *children’s* needs: “Once we have a spectrum of interventions that kids can move along very easily, we’ll be in a much better position.”

*Parent:* The big issues going forward are maintaining the institutional memory of how the services evolved to where they are now, improving on those services, and addressing that this is a spectrum disorder and it’s a continuum issue, from diagnosis to death. The needs change. With my own son, we’ve seen the difference in what he required when he was 10 as opposed to when he’s 18. We’re not going to be here forever, so we want to be confident that there’s appropriate housing and care in place for him.

*Policymaker:* Autism is a lifelong disability. There needs to be a broad range of those services and supports available across the entire continuum because people are going to be in different places at different times throughout their lives.

*Researcher:* If everybody would keep their eye on the prize—the kids—people would be able to work together way better. The kids are the ones who I think can bring all of those groups together more, to really understand what it means to make a difference in a kid’s life and why it’s important.

#### Doing More for All Children

For most participants, this call for more comprehensive services extended beyond children with autism—encompassing children with other mental health and developmental difficulties: “I don’t want to take away from kids with autism, but there has to be something to widen the possibilities.” Some participants even suggested that autism could serve as a model for making better collective decisions on behalf of *all* children.

*Parent:* I have mixed feelings about it because I deal with other families who don’t have that autism diagnosis and they don’t have any hope of having any help, so it’s an unfair policy to others who desperately need help as well. But you still can’t ignore what’s happening with autism, with so many children receiving this diagnosis now.

*Policymaker:* So, parents of children with autism have forced us to take them into account in developing and delivering services. They’re a voice that needs to be heard and understood. If the parents of other children who are at-risk do not have the same ability to be heard, then we should be paying attention to them anyways, and we should make sure that we make our decisions around the services we offer based on what their needs are, whether they recognize them or not.

*Researcher:* There’s a backlash right now in society. People are resentful that so much money’s being spent on autism treatment. Why should we have special treatment for children with autism when there are other kids with developmental disorders for whom we don’t have specialized treatment programs? My take is, well, I don’t think we should *not* do something for some children because we can’t do it for *all*. I think we should be trying to do more for all.

## Discussion

Canadian autism policy has been characterized by intense acrimony, potentially hindering progress on improving children’s services. We therefore wanted to understand the conflicts and to ascertain whether consensus was possible. Parents vividly recounted first learning of their child’s autism, then struggling to find supports and services, all while meeting extraordinary parenting demands. They described having to become dedicated champions for their own children, with many going on to attempt to influence policy for all children with autism, often with support from researchers. Policymakers were sympathetic to these parents, but in turn, struggled with meeting the needs of all children in the population. Meanwhile, all participants agreed on the need for a more comprehensive approach to autism services—*across the spectrum* and *throughout the lifespan*. So while we encountered diverse views and experiences, we also found an emerging consensus among parents, policymakers and researchers on the necessity of diversifying services for children across the autism spectrum, providing greater support during the transitions into school and into adulthood, expanding existing services to reach more disadvantaged children and families, and increasing public investments in children’s mental health and development more broadly.

We believe that our sample captured a reasonable cross-section of those who have engaged in the autism policy debates in Canada to date, and that our qualitative methods allowed participants to explore the reasons for the conflicts and the opportunities for agreement in considerable depth. Nonetheless, one important limitation needs highlighting. We did not include young people with autism, whose perspectives may differ from those of parents, policymakers and researchers (Orsini and Smith 2010). Our goal was to understand how the Canadian policy process for autism services has unfolded since the late 1990s. However, children with autism have been largely excluded from this process—indeed, children are often excluded from decision-making about the very services that are intended to help them. To the fullest extent possible, young people with autism should therefore be included in future policy deliberations to better understand how we may collectively meet their needs (Mottron 2011).

Although most provinces have increased EIBI investments in response to parent advocacy, participants still called for a more comprehensive range of interventions for children across the autism spectrum. These findings lend support to ongoing efforts to develop and evaluate new interventions in real-world settings (Dingfelder and Mandell 2011; Smith et al. 2010). Ideally, a wider array of interventions will also better accommodate the increasingly apparent heterogeneity in children’s developmental trajectories (Fountain et al. 2012; Georgiades et al. 2013). Furthermore, most parents in this study favoured pragmatic approaches to help their children cope day-to-day and develop independence, thereby enhancing their life chances (Brown et al. 2012; Pituch et al. 2011).

We also found widespread disquiet among all participants over the lack of services and supports for school-aged children and adolescents. These findings underscore the need for more effective school-based interventions for young people with autism (Kasari and Smith 2013). Even more poignantly, all participants described a dearth of supports and services for young adults with autism. Our findings therefore echo concerns expressed by others about the need for greatly expanded service capacity throughout the lifespan, making the transition to adulthood a particularly high priority for new research and policy investments (Cadman et al. 2012; Shattuck et al. 2011).

Despite the past conflicts, we also found evidence for emerging consensus among parents, policymakers and researchers on the need for much more comprehensive autism services—a consensus that could inform future “collective ethical judgments” for children. It will take concerted efforts to realize this aim, given the “fading of the redistributive state” in Canada, where new public investments are almost always “timid and selective in terms of the broader population of the needy” (Banting and Myles 2013, p. 334). However, it is worth recalling that parents of children with autism have already had a remarkable influence on policymaking in Canada, despite the extraordinary burdens that these children and their families carry. These parents are exemplars for influencing policy. Yet no parent should have to resort to such extraordinary measures to obtain essential services and supports for their children. Stark service shortfalls exist for many other Canadian children, too, particularly those with other mental health and developmental difficulties (Waddell et al. 2014). Perhaps most importantly then, our findings suggest that more public resources should be made available to adequately support *all* children with mental health and developmental difficulties. In the words of one of our participants: “We should be trying to do more for all.”
